# DETERMINANTS AND TRAJECTORIES OF HEALTHY AGING IN OLDER AMERICANS

**DOI:** 10.1093/geroni/igae098.2400

**Published:** 2024-12-31

**Authors:** Lujain Sahab

**Affiliations:** King’s College London, Liverpool, England, United Kingdom

## Abstract

**Background:**

As the proportion of ageing individuals in the population increases, there is an increase in the burden of age-related chronic conditions. Therefore, it is essential to identify determinants of healthy ageing through extensive research and implement strategies to address challenges in society.

**Methods:**

This analysis included an average of 8,340 participants, aged 50+ years, who participated in 6 waves (2006-2016) of the Health and Retirement Study. Healthy ageing measurement included freedom from cognitive impairment, freedom from disability, and high physical functioning. The study examined socioeconomic and ethnic inequality in healthy ageing over 10 years. Multilevel analysis was used to assess education, wealth, income and ethnic inequality in health ageing while accounting for demographic and behavioural factors.

**Results:**

The analysis found that Females had lower odds for healthy ageing (OR: 0.64, 95% CI: 0.61–0.67). Black individuals had statistically significant lower odds of ageing healthily (OR: 0.87, 95% CI: 0.81–0.93) while hispanics had higher odds. The higher the educational degree the more likely you are to age healthily. Former smokers have a higher chance of healthy ageing (OR: 0.84, 95% CI: 0.80–0.88). The higher an individual’s wealth and income the more likely they are to age healthily. People who reported their health as poor were more likely to age negatively (OR: 0.22, 95% CI: 0.20–0.23).

**Conclusion:**

This study showed that Being male, white, wealthy, with a high income were associated with a higher chance of achieving healthy ageing.

